# Analysis of DNA methylation of E‐cadherin and p16^ink4a^ in oral lichen planus/oral lichenoid lesions

**DOI:** 10.1002/cre2.355

**Published:** 2020-12-03

**Authors:** Takatoshi Chujo, Koki Yoshida, Rie Takai, Osamu Uehara, Hirofumi Matsuoka, Tetsuro Morikawa, Jun Sato, Itsuo Chiba, Kenichi Matsuzaka, Yoshihiro Abiko

**Affiliations:** ^1^ Department of Pathology Tokyo Dental College Tokyo Japan; ^2^ Division of Oral Medicine and Pathology, Department of Human Biology and Pathophysiology, School of Dentistry Health Sciences University of Hokkaido Sapporo Japan; ^3^ Advanced Research Promotion Center Health Sciences University of Hokkaido Sapporo Japan; ^4^ Division of Disease Control and Molecular Epidemiology, Department of Oral Growth and Development, School of Dentistry Health Sciences University of Hokkaido Sapporo Japan

**Keywords:** DNA methylation, E‐cadherin, oral lichen planus, p16

## Abstract

**Objectives:**

Epigenetic phenomena are changes in gene expression not involving the DNA sequence. DNA methylation is a major occurrence underlying epigenetic changes in human cells. Although aberrant DNA methylation is well documented in malignant lesions, limited information has been shown on the involvement of DNA methylation in oral lichen planus and oral lichenoid lesions (OLP). The present study aimed to investigate DNA methylation of E‐cadherin and p16 in OLP, and compare the findings with those in non‐inflamed gingiva (Non), radicular cyst (RC), and oral squamous cell carcinoma (SCC).

**Materials and methods:**

Paraffin‐embedded surgical biopsy specimens were sliced, DNA was extracted, bisulfite treatment was applied, and methylation‐specific polymerase chain reaction was performed. Immunohistochemistry was performed to observe the relative expression patterns of these genes.

**Results:**

E‐cadherin was hypermethylated in OLP (*p* < 0.01), SCC (*p* < 0.01), and RC (*p* < 0.05), when compared with Non; DNA hypermethylation was confirmed in OLP and SCC when compared to Non and RC. Hypermethylation of p16^ink4a^ was observed only in SCC (*p* < 0.01).

**Conclusion:**

DNA methylation levels of E‐cadherin and p16^ink4a^ were significantly higher in OLP than in normal tissues, and may be associated with the pathogenesis and progression of the disease.

## 1 INTRODUCTION

Oral lichen planus and oral lichenoid lesions (OLP) is a chronic inflammatory disease that involves the oral mucosa (Al‐Hashimi et al., 2007). The precise etiology of OLP is unknown; genetic background, systemic associations, and environmental factors such as dental metals, drugs or viral infections have been involved in OLP (Alrashdan et al., 2016). Environmental factors are known to cause epigenetic modifications, wherein only the pattern of gene expression is altered but not the DNA sequence. Alterations in DNA methylation form a crucial part of epigenetic modification (García & García‐García, 2012; Jithesh et al., 2013; Qiu et al., 2004). Oral cancerous and precancerous lesions have shown aberrant epigenetic modifications, including hypermethylation of tumor‐related genes (Castilho et al., 2017; Hema et al., 2017; Lingen et al., 2011) such as E‐cadherin and p16^ink4a^ (Castilho et al., 2017; Hema et al., 2017; Lingen et al., 2011). E‐cadherin is a 120 kDa glycoprotein in a part of the adhesive junctions and functions as a tumor suppressor gene. The hypermethylation of E‐cadherin followed by the downregulated expression of this gene leads to malignant transformation and tumor progression (Burassakarn et al., 2017). The p16^ink4a^ protein functions as a negative regulator of cell cycle progression via inhibition of both cyclin‐dependent kinase (CDK) 4 and 6 followed by Rb cyclin‐dependent phosphorylation (Montebugnoli et al., 2011). The reduction of p16^ink4a^ promotes cell growth and often contributes to malignant transformation (Okuma et al., 2017). In OLP, aberrant immunohistochemical staining for both E‐cadherin and p16^ink4a^ have been reported (Ebrahimi et al., 2008; Lee et al., 2016; Montebugnoli et al., 2011; Sridevi et al., 2015); however, the mechanisms involved in these phenomena remain unknown. Herein, we hypothesized that E‐cadherin and p16^ink4a^ hypermethylations may cause the aberrant expression of these proteins, based on which, we examined DNA methylations in the promoter regions of E‐cadherin and p16^ink4a^ in OLP, and compared them with those in non‐inflamed healthy gingiva (Non), radicular cyst (RC), and oral squamous cell carcinoma (SCC).

## 2 MATERIALS AND METHODS

### 2.1 Samples

Biopsy samples embedded in paraffin and surgical materials of OLP (*n* = 26), RC (*n* = 31), SCC (*n* = 25), and Non (*n* = 25) were used in this study. The OLP samples were collected by the following histopathological diagnostic criteria; hyperkeratosis, parakeratosis, basal cell hydropic change, Civatte bodies, a band‐like lymphocytic infiltrate in the lamina propria, and excluding the presence of dysplasia or malignant tumor (Al‐Hashimi et al., 2007; Cheng et al., 2016). The study was approved by the ethics committee of Institute of Personalized Medical Science, Health Sciences University of Hokkaido (No. 2012–005). Paraffin sections were prepared with a microtome and used for methylation‐specific PCR (MSP) and immunohistochemistry.

### 2.2 Quantitative methylation specific PCR assay

DNA methylation levels in CpG islands were analyzed using the Quantitative methylation‐specific PCR (qMSP) assay. Genomic DNA was extracted from paraffin sections using the Epitect Plus FFPE Lysis kit (Qiagen, Venlo, Netherland). The DNA samples were treated with sodium bisulfite using the EpiTect Plus Bisulfite Kits (Qiagen). DNA methylation of E‐cadherin and p16^ink4a^ genes were analyzed by SYBR green‐based qMSP. The methylated and unmethylated primers were designed as described in a previous study (Table 1) (Herman et al., 1996). PCR was performed in a total volume of 25 μl, with 1.0 μl of bisulfite‐treated DNA template mixed with 12.5 μl of KAPA SYBR FAST qPCR Kit (Nippon Genetics, Tokyo, Japan) and a pair of primers at a final concentration of 400 nM. The PCR conditions included initial incubation at 50°C for 2 min, denaturation at 95°C for 10 min, 40 cycles of denaturation at 95°C for 15 s, and annealing at 58°C for 1 min. After PCR amplification, a dissociation curve was generated to confirm the size of the PCR product. The percentages of E‐cadherin and p16^ink4a^ methylation in a sample were estimated using the following formula (Lu et al., 2007): Methylation (%) = $\frac{M}{M+U}\times 100=\frac{1}{1+\frac{U}{M}}\times 100=\frac{1}{1+2^{\left(-\Delta \mathrm{Cq}\right)}}\times 100$, where *M* and *U* are the copy numbers of methylated and unmethylated E‐cadherin/ p16^ink4a^, respectively, and Δ*Cq* = *Cq*_*U*_ − *Cq*_*M*_.

**TABLE 1:** Primer sequences (5′ to 3′)

Gene	Forward	Reverse	Product length (bp)	(Refs.)
E‐cadherin: M E‐cadherin: U	TTAGGTTAGAGGGTTATCGCGT TAATTTTAGGTTAGAGGGTTATTGT	TAACTAAAAATTCACCTACCGAC CACAACCAATCAACAACACA	116 97	Lingen et al. (2011) Lingen et al. (2011)
p16^ink4a^: M p16^ink4a^: U	TTATTAGAGGGTGGGGCGGATCGC TTATTAGAGGGTGGGGTGGATTGT	GACCCCGAACCGCGACCGTAA CAACCCCAAACCACAACCATAA	150 151	Lingen et al. (2011) Lingen et al. (2011)

Abbreviations: M, methylation primer; U, unmethylation primer.

### 2.3 Immunohistochemistry

Immunohistochemical localizations of E‐cadherin and p16^ink4a^ were observed in the tissue sections. The slides were deparaffinized in xylene (3 changes for 3 min each) and rehydrated in a series of graded alcohol (100, 90, 80, and 70% each for 3 min). Endogenous peroxidase activity on the slide was blocked with 3% H_2_O_2_ in methanol for 10 min. Mouse Anti‐Human E‐cadherin monoclonal antibody (DAKO, Tokyo, Japan) and rabbit anti‐clone EPR1473 p16 monoclonal antibody (DAKO) were used as primary antibodies. EnVision + system‐HRP‐labeled polymer anti‐mouse and anti‐rabbit were used as secondary antibodies (DAKO). Reaction products were visualized with diaminobenzidine chromogen concentrate (Dako), and finally counterstained with hematoxylin. Immunostaining was graded as − (<10% positively stained cells), ± (10%–25% positively stained cells; weak expression), + (25%–50% positively stained cells; mild to moderate expression), and ++ (50%–100% positive cells; moderate to strong expression).

### 2.4 Statistical analysis

Statistical analysis was performed on a database using the IBM SPSS Statistics 23 (IBM, Armonk, NY). Results were compared using the Kruskal–Wallis test. Data are presented as means ± standard deviation (SD), and significance levels were set at ^*^ *p* < 0.05 and ^**^ *p* < 0 .01 when compared with the controls.

## 3 RESULTS

**FIGURE 1:**
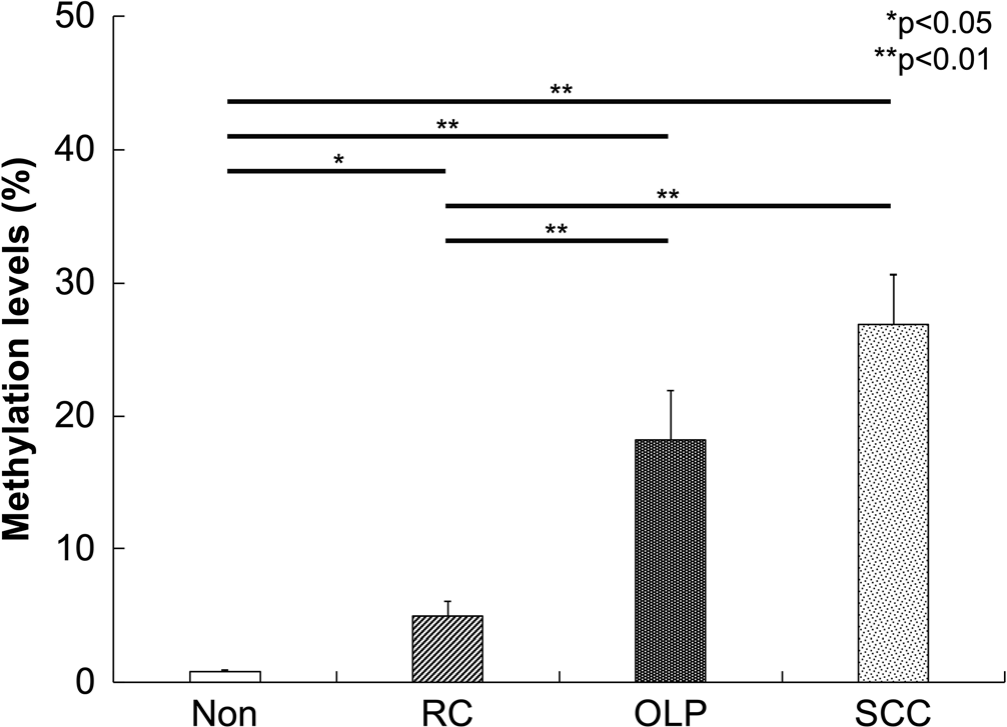
The percentage of DNA methylation of E‐cadherin as measured by qMSP. DNA methylation levels of E‐cadherin were significantly higher in OLP than in Non (*p* < 0 .01) and RC (*p* < 0.05), whereas no significant difference was found between OLP and SCC. ^*^ *p* < 0.05, ^**^ *p* < 0.01

**FIGURE 2:**
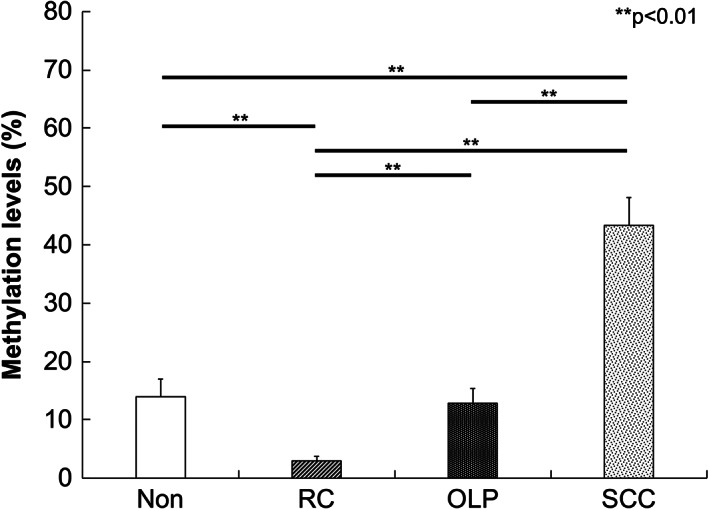
The percentage of DNA methylation of p16^ink4a^ as measured by qMSP. DNA methylation levels of p16^ink4a^ were significantly higher in OLP than in Non and RC (*p* < 0 .01), and significantly lower in OLP than in SCC (*p* < 0.01). ^**^ *p* < 0.01

DNA methylation levels of E‐cadherin were significantly higher in OLP than in Non and RC (*p* < 0.05), whereas no significant difference was found between OLP and SCC (Figure 1). Similarly, significantly higher levels of p16^ink4a^ DNA methylation were observed in OLP when compared with both Non and RC (*p* < 0.05); on the other hand, the methylation level in OLP was significantly lower than that in SCC (*p* < 0.05, Figure 2).

**FIGURE 3:**
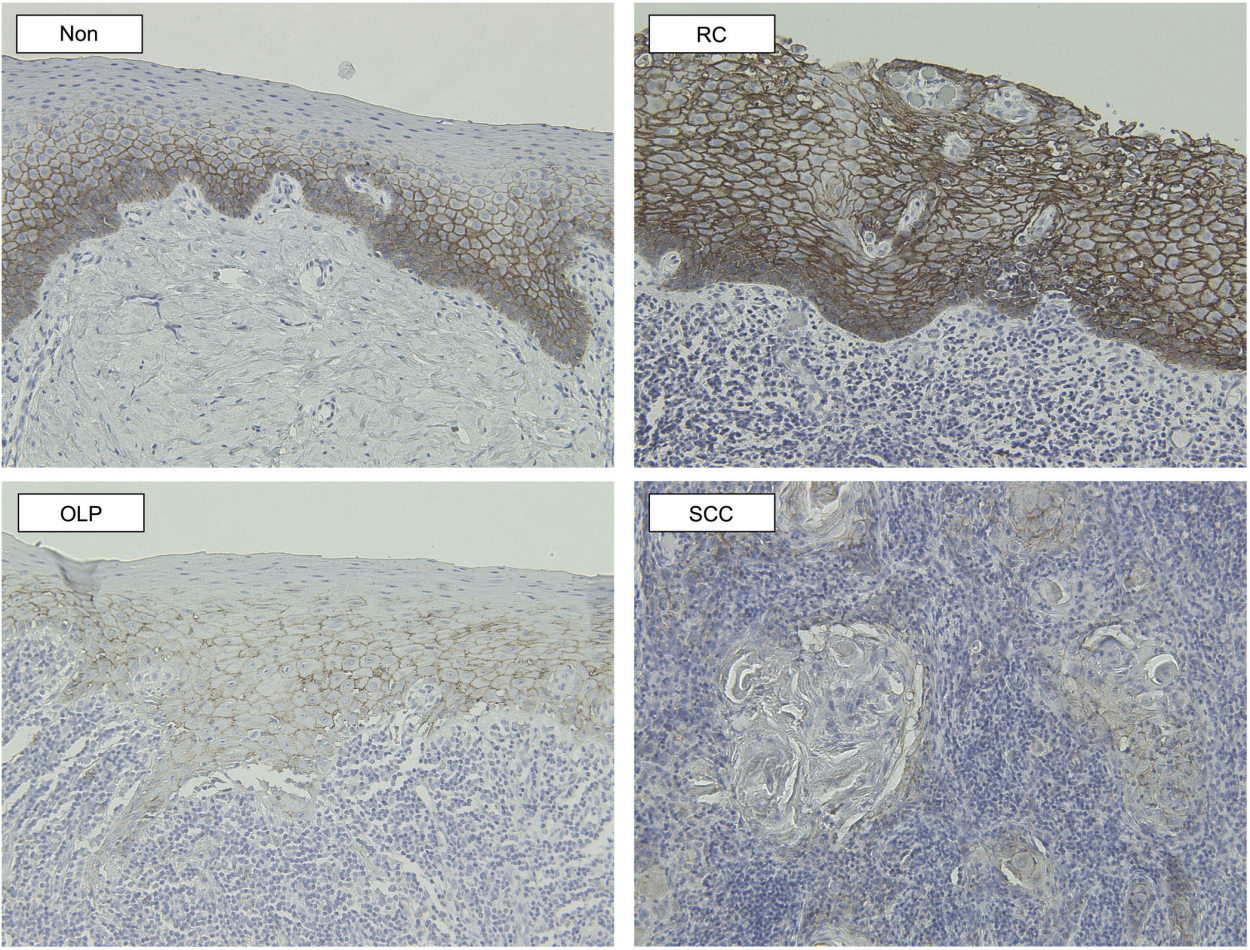
Immunohistochemical staining for E‐cadherin (magnification, ×200). Immunohistochemical staining for E‐cadherin was mainly localized at the intercellular contact areas in the basal to spinous layers in OLP. No positive staining was observed in the keratinized layers. The positive reactions were stronger and more extensive in Non and RC when compared to OLP. The staining grades were ++ in Control and RC, and + in OLP and SCC

**FIGURE 4:**
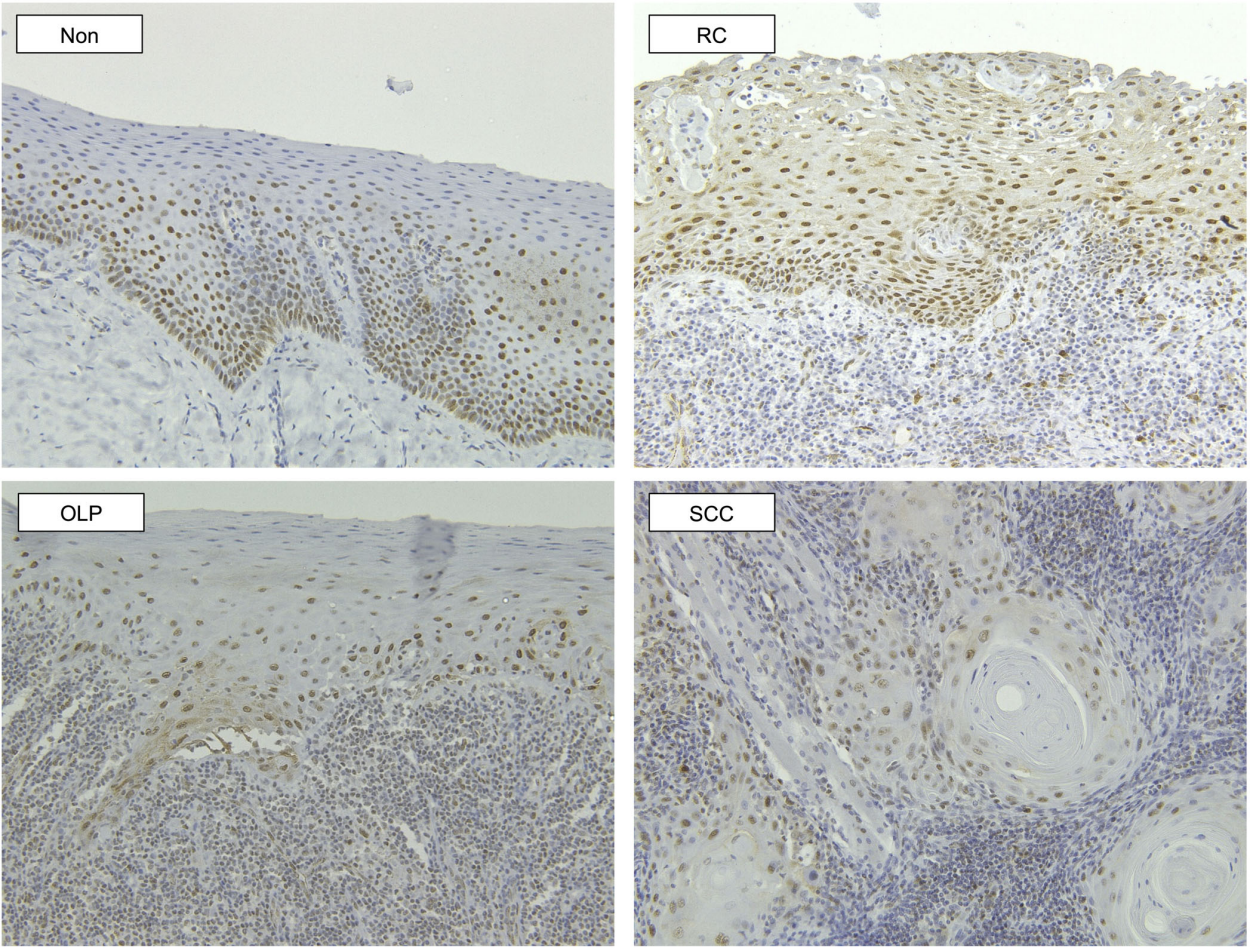
Immunohistochemical staining for p16^ink4a^ (magnification, ×200). In OLP, immunohistochemical staining for p16^ink4a^ was observed in the nuclei of cells in the basal and spinous layers. The positive reactions were more widespread in Non than in OLP. The grades of positive staining were ++ in RC and + in OLP, Non, and SCC

Immunohistochemical staining for E‐cadherin was mainly localized at the intercellular contact areas in the basal to spinous layers in OLP. No positive staining was observed in the keratinized layers. The positive reactions were stronger and wider in Non and RC than in OLP, and the grades were as follows: ++ in Control and RC, and + in OLP and SCC (Figure 3). Immunohistochemical staining for p16^ink4a^ was observed in the nuclei of the cells in the basal and spinous layers in OLP. The positive reactions were wider in Non than in OLP, and were graded as ++ in RC and + in OLP, Non, and SCC (Figure 4).

## 4 DISCUSSION

In the present study, we examined the DNA hypermethylation of E‐cadherin and p16^ink4a^ in OLP, and compared the results with those in non‐inflamed epithelial tissues (Non), non‐specific inflammatory epithelial tissues obtained from RC, and oral SCC. The methylation levels of E‐cadherin in OLP were higher than that in Non and RC, and similar to that in SCC. The methylation levels of p16^ink4a^ in OLP were significantly higher than that in Non and RC, but lower than that in SCC. Thus, hypermethylation of both E‐cadherin and p16^ink4a^ may be observed in OLP.

Hypermethylations of E‐cadherin and p16^ink4a^ have been reported in malignant tumors, including oral SCC (Viswanathan et al., 2003). The hypermethylation of E‐cadherin followed by a downregulation in its expression levels leads to malignant transformation and tumor progression (Pannone et al., 2014; Schmalhofer et al., 2009). The reduction of p16^ink4a^ promotes cell growth and often contributes to malignant transformation (Kato et al., 2006). Although E‐cadherin hypermethylation in OLP not been demonstrated so far, aberrant immunohistochemical staining of E‐cadherin was reported (Du & Li, 2015). Loss of membrane expression and decreased expression levels of E‐cadherin were observed in OLP (Sridevi et al., 2015). Consistent with the previous reports (Sridevi et al., 2015), the results of the current study showed a significantly lower expression of E‐cadherin in OLP when compared to that in Non or RC. However, these reports did not state the mechanism involved in the aberrant immunohistochemical expression. Hypermethylation results in the downregulated expression of the gene. We believe that hypermethylation may cause the aberrant expression of E‐cadherin, which has been linked to malignancy in OLP (Du & Li, 2015). Thus, the involvement of aberrant E‐cadherin expression in the pathogenesis of OLP should not be ruled out. Metal allergy is one of the environmental factors associated with the etiology of oral lichen planus/oral lichenoid lesions (Suter & Warnakulasuriya, 2016), and may be associated with that of OLP, although the precise etiology of OLP remains unknown. A previous study showed E‐cadherin hypermethylation induced by nickel in bronchial epithelial cells (Wu et al., 2012). Thus, other metals similar to nickel may directly cause OLP via hypermethylation of E‐cadherin. Nonetheless, additional researches are needed to clarify this speculation.

Frequent methylation of p16^ink4a^ in OLP has been reported recently (Dang et al., 2013) wherein p16^ink4a^ levels in OLP patients were found to be higher than that in heathy subjects, and lower than that in SCC. These findings are similar to those observed in the present study. Aberrant immunohistochemical staining for p16^ink4a^ has also been demonstrated previously (Nasser et al., 2011) with increased staining in OLP when compared to normal controls (Poomsawat et al., 2011). A previous paper suggested that the positive staining for p16^ink4a^ in OLP is associated with eactive inflammatory processes rather than the progression risk to malignant tumor (Montebugnoli et al., 2011). The immunohistochemical findings of the current study showed increased positive staining in RC than in OLP and Non. Although our results were not in accordance with those reported previously, the increased positive reactions for p16^ink4a^ observed in RC may support the suggestion that p16^ink4a^ staining is related to reactive inflammatory processes. However, the mechanism by which p16^ink4a^ hypermethylation is involved in its aberrant expression needs to be investigated in future.

In conclusion, the present study demonstrated that the methylation levels of E‐cadherin and p16^ink4a^ in OLP were significantly higher than that in normal tissues suggesting that the hypermethylation of these genes may be related to the pathogenesis and progression of OLP. In addition, epigenetic modifications due to environmental factors may also be involved in the pathogenesis of OLP. However, further investigations are needed to prove this hypothesis.

## CONFLICT OF INTEREST

The authors declare that they have no conflict of interest.
